# Designing a model of indifference in theorizing in management research with grounded theory approach

**DOI:** 10.3389/frma.2024.1460135

**Published:** 2024-12-13

**Authors:** Maryam Asgharinajib, Sima Aalipour, Shahryar Sorooshian

**Affiliations:** ^1^Saito University College, Selangor, Selangor, Malaysia; ^2^Department of Business Administration, University of Gothenburg, Gothenburg, Sweden

**Keywords:** theorizing, management, indifference in theorizing, scientific silence, grounded theory, Iran

## Abstract

This study explores and elucidates the phenomenon of indifference in theorizing within management research in Iran, highlighting the causal conditions and implications of this indifference on both the academic and practical landscapes of management. Using a qualitative grounded theory methodology, this study synthesized the data collected through interviews with management faculty members from various Iranian universities. Purposive sampling was employed to select participants until theoretical saturation was achieved with 29 interviews. The data were analyzed using ATLAS.ti software, enabling the construction of a paradigm model to explain the observed phenomena. The study identified multiple causal conditions contributing to indifference in theorizing, including individual, educational, cultural, economic, social, political, and systemic factors. These factors collectively foster a climate of scientific isolation, hindering the development of management theories and affecting both educators and students. The outcomes of this indifference manifest as a reduction in theoretical innovation and diminished engagement with management theory among academics and practitioners. Indifference, as a key concept in the presented model, represents a type of scientific silence, indicating the unwillingness or inability of researchers to create new and effective theories in the field of management. This research contributes to the field by providing a detailed model of the dynamics underlying indifference in theorizing within management studies in Iran, a topic that has received limited attention in the existing literature. The study's findings emphasize the need for systemic changes to overcome barriers to theorizing and suggest pathways for revitalizing theoretical contributions in management research.

## 1 Introduction

There is a growing body of literature in management on the theorizing process, i.e., how theories are constructed. This growing body of literature offers many tools and approaches for theorizing (Mollah, 2019). However, there is no coherent understanding of how these tools fit together. For example, when to use a specific tool and what combination of tools can be used in the theorizing process (Shepherd and Suddaby, 2017). A systematic review of the literature related to theorizing in management integrates the various individual components of theorizing into a coherent whole. The importance of narrative or storytelling in theorizing becomes apparent when reading this growing body of literature (Pollock and Bono, 2013; Van Maanen, 1995). A community of researchers may adopt a theorizing approach that uses empirically inspired, interesting findings about management phenomena to inform and refine initial guesswork. They also consider the reason behind our desire for science and theory (Golshani, 2016). Theory creation is usually motivated by a desire to explain something. Before a theory can be developed, there is usually a theoretical problem. But where does the theoretical problem come from? Theoretical issues can arise from a desire to accomplish certain goals, concerns about the social effects or social consequences of something, or a desire to better understand some process.

Through theorizing, researchers can uncover anomalies and highlight the need for theory by accurately identifying trends. Hence, organization and management researchers regularly generate new theoretical insights as they respond to changes in management practices (Sumpter et al., 2021). Technology, globalization, and social trends also cause change, which means that organizations and work are dynamic. Ideally, the dynamism of the theoretical structure would align with the dynamic nature of today's organizations. However, with the evolution of management and organizational theories, researchers should also remain attentive to established paradigms within their research fields (Sumpter et al., 2021). Thus, consensus on common theoretical constructs is essential for fostering scientific progress (Cole, 1983). Therefore, management science researchers have paid considerable attention to the role of theory. The precondition for publication in influential management journals is that articles must contribute theories (Colquitt and Zapata-Phelan, 2007; Hambrick, 2007; Rynes, 2005; Sutton and Staw, 1995). Some researchers question the prominence of theory (Hambrick, 2007; Pfeffer, 2014) and debate whether theory building in the social and organizational sciences has adhered to the goal of rejecting accepted views in an attempt to build new theories. The wisdom inherent in citation is tied to a very narrow view of the nature of the theory-building process itself (Gioia and Pitre, 1990). Traditional approaches to theorizing in organizational studies tend to produce valuable, yet incomplete, views of organizational knowledge, largely because they are based on the tenets of a core paradigm (Kuhn, 1970) or a particular way of understanding organizational phenomena (Gioia and Pitre, 1990). Some researchers believe that there is not much debate about the importance of building theories for the advancement of management knowledge (Suddaby, 2014). For example, scholars demand new theories in areas such as organization (Suddaby et al., 2011), entrepreneurship (Shepherd, 2015), management (Barkema et al., 2015), and work (Okhuysen et al., 2013).

Theorizing is important for the advancement of management knowledge (Shepherd and Suddaby, 2017), and understanding how theory is constructed or arises is an essential part of knowledge production for all researchers (Philipsen, 2018). This is especially true in Iran, where organizations face different challenges and opportunities from other countries due to political issues, including international sanctions. Theorizing is important in management because theory building is inherently related to problem-solving in real-world scenarios. The interplay between social values and scientific research suggests that management theories developed in one cultural context may not be directly applicable to another context (Bagheri, 2009). In addition, since management theories originated in the West, management practices in Iran require localized theories that are needed for application to address real-world practical problems. Therefore, the production of management knowledge will affect the country's future, based on its specific management needs. The creation of these theories and their application in practice have become a concern (Danaeifard, 2009). Hence, this study examined the factors that contribute to this issue and the effects it has on both the theoretical and applied fields of management, with the objective of better understanding the phenomenon of indifference in theorizing in Iranian management research and answering the question: Which theory explains indifference in theorizing in management research?

## 2 Methodology

The current study used the grounded theory strategy, which is a type of qualitative strategy, because the grounded theory method, in addition to being used in theorizing, is considered a set of implementation techniques in qualitative research (Corbin and Strauss, 2014). A systematic approach was used among various data theory approaches. Since interviews serve as the most popular method of data collection for grounded theory (Corbin and Strauss, 2014), the main source of data collection for this study was also interviews.

To collect data, in-depth semi-structured interviews were conducted with management faculty members at universities, who were selected using purposive sampling. Three criteria were established for the selection of participants in the study: (1) Expertise and experience: Individuals with expertise related to the research topic were selected, which included university professors and researchers who had teaching or research experience in the field of research. For this criterion, a teaching experience of more than 10 years and the publication of more than 10 articles related to the research topic were taken into consideration. (2) Educational qualification: Individuals with a relevant academic qualification (such as a doctorate or master's degree) were selected because it indicated the depth of one's knowledge and experience in the subject area. (3) Diversity of views: Professors from various universities in Iran were invited to participate in the interviews to obtain more comprehensive results regarding the field of research. According to the main question of the research, “theory explains indifference in theorizing in management research?”, the data collection approach began with the following four questions, which subsequently led to more comprehensive open discussions informed by the participant responses. This approach aimed to generate fresh concepts and patterns that could be disregarded when employing focused inquiries:

1. What is the importance of theorizing in the field of management in Iran?
2. What are the educational and research barriers that researchers face in theorizing in the field of management in Iran?
3. What are the environmental obstacles that researchers face in theorizing in the field of management in Iran?
4. What are the reasons for the lack of applied theories in the field of management in Iran?

After exchanging information via text messages, an interview session was scheduled with the participants. Some of the interviews were conducted face-to-face, while others were conducted via video call on Skype due to geographical restrictions. The average time of the interviews was 45 min (ranging from 38 to 73 min). At the beginning of the interview, the objectives of the study were reviewed. After the participants consented to participate and allowed the meeting to be audio-recorded, data collection began with demographic information and continued with an in-depth semi-structured interview. The interviews continued until theoretical saturation was reached, which occurred after 29 interviews. A total of 62% of the participants in this study were male, 38% were female, 46% were full professors, and 54% were associate professors.

Since in systematic grounded theory, data analysis is integrated throughout the entire process (Corbin and Strauss, 2014), the process of data analysis began immediately after the first interview, through writing notes and memos. After each interview, this process continued. Constant comparative analysis also began with the first collected data and continued throughout the entire research process. As information developed and data began to be integrated, the notes led to open coding, axial coding, and selective coding (Corbin and Strauss, 2014). The ATLAS qualitative analysis software (ATLAS.ti) was also used to enhance the data analysis.

## 3 Results

In this section, the findings obtained from the data analysis are presented based on the grounded theory method, which led to the identification, summarization, classification, and creation of elements located in the realm of indifference in theorizing, after conducting in-depth interviews with the experts.

### 3.1. First step: open coding

Primary coding: At this stage, after listing all the key points of the interviews, a code was assigned to each point. Table 1 shows the primary codes extracted from the interviews.

**Table 1:** Primary codes extracted from the interviews.

**Interview text (key points)**	**Open coding**
Teaching the philosophy of science can be very effective in generating and creating new ideas. In fact, the lack of philosophy education in graduate management courses is one of the biggest gaps that education policy centers have ignored by creating research-oriented policies	Failure to pay attention to teaching the philosophy of science in postgraduate courses
We need to direct our research toward problem-oriented research. In fact, the identification of existing issues and conversations can create a platform to stimulate theorizing	Necessity of problem-oriented research
The time-consuming process of accepting articles and sometimes partisanship in accepting articles reduces the motivation of researchers	Partisanship in accepting articles and reduced motivation
The failed and traditional system governing universities links the promotion of faculty members to the number of their articles	Promotion of faculty members according to the number of articles
Criticism of scientific research should be done in such a way that the character of the researcher is not destroyed, as this matter will have no other result than a famine of discourse and conflict of opinions	Inappropriate and destructive approach to judging and evaluating research
The mental conflicts of professors and students regarding livelihood concerns, due to inflation and exchange rate instability, are another obstacle that prevents them from focusing on scientific research	Livelihood concerns, inflation, and exchange rate instability in Iran

Secondary coding and formation of the main categories and subcategories: In the next step, the primary codes were converted into secondary codes. Table 2 shows the results of the open coding, based on the secondary code, main categories, and subcategories.

**Table 2:** Main categories and subcategories extracted along with the secondary codes.

**Main category**	**Subcategory**	**Secondary code**
Educational factors	Lack of proper methodology	• Lack of familiarity with research and methodology • Lack of attention to research training • Lack of familiarity with various methodologies and their non-generative nature • Lack of importance of methodology in the scientific productions of the field of management
	Weakness of graduate education in the field of management	• Lack of management knowledge in the country • Lack of awareness of current knowledge in the field of management • Necessity of a suitable educational and training program in the field of management • The rule of the traditional way of education • Lack of attention to the philosophy of science education in the post-graduate course
	Inadequate needs assessment for research and its tools	• A close bond between the researcher and the subject • Lack of problem-oriented research • Lack of access to existing knowledge references
Social factors	Lack of development of critical thinking	• Lack of interest in management criticism and debate chairs • Failure to hold Delphi and brainstorming sessions in universities • Weakness in discussing management theories
	Conflict in cyberspace and increasing availability of entertainment	• Reducing the opportunity for reflection and critical thinking in theoretical fields due to the introduction of technology • Receiving a large amount of information through the internet, leading to confusion among researchers
Cultural factors	The influence of the environment and collective opinions	• Xenophobia • Sacredness of some scientific texts • Personality worship • Halo error • Stereotyping • Structural convergence
Systemic factors	Bureaucratic pressures governing the atmosphere of universities	• Rules and regulations and pure obedience • Cumbersome rules and regulations • Absence of proper placement away from orders and directives
	Increasing the formal production of science	• Focus only on the quantity of articles • Necessary to show the number of articles, not the intellectual connection with the findings • Essay-oriented universities • Promotion of faculty members according to the number of articles • Reducing the opportunity for master's and doctoral students to benefit from the cooperation of faculty members on a per capita basis
	Defective system of evaluation and acceptance of articles	• Inappropriate approach to judging and evaluating research • Stereotype criteria for the acceptance of articles
Interdisciplinary factors	Pure quantification	• Dominance of quantitative methods • Dominance of technical and objective approach in studies
	The nature of management	• Human centrality in the subject of management studies • Complexities of research subjects, especially the complexity of organizations
	Disregarding the wisdom of fields	• Lack of connection between the previous possessions and the current potential • Enmity toward the science of foreign management • False belief that science means experimental science • Avoiding extremes in accepting imported scientific knowledge
Political factors	Not prioritizing thought and critical thinking	• Failure to value professors and researchers and maintain the scientific and social base of professors • Failure to formulate cooperation policies for organizations • Lack of communication between professors and management students and organizations and businesses • Failure to create a practical space for presenting management science
Economic factors	Financial problems	• Livelihood concerns • Inflation • Exchange rate instability in Iran
	Lack of job security	• Student unemployment • Job stress
Individual factors	Personality	• Self-control • Irresponsibility • Not decisive
	Attitudinal	• Relative compatibility with the system • Discouragement of students and professors • Task orientation
Indifference in theorizing	Suppressing the courage to comment	• Fear of theorizing • Promoting adaptationism and conservatism • The trap of addiction to common theories • Lack of scientific self-confidence
	Lack of open theorizing processes	• Lack of sharing of concepts, frameworks, theoretical relationships, and case examples • Collective action problems
Outcomes	Implications for management professors and students	• Indifference of management students and professors toward progress • Reducing the spirit of commitment to theorizing • Weakening the spirit of research • Tired of theorizing • Decreased motivation • Individual alienation
	Outcomes related to the management education system	• Mental stagnation • Lack of indigenous theories of management • Lack of management growth in Iran • Lack of scientific attitude to management
Actions and reactions	Scientific isolation	• Research indifference • Reduced research interactions • Separation of professors and students from the scientific body of management

### 3.2 Second step: axial coding

**Figure 1:**
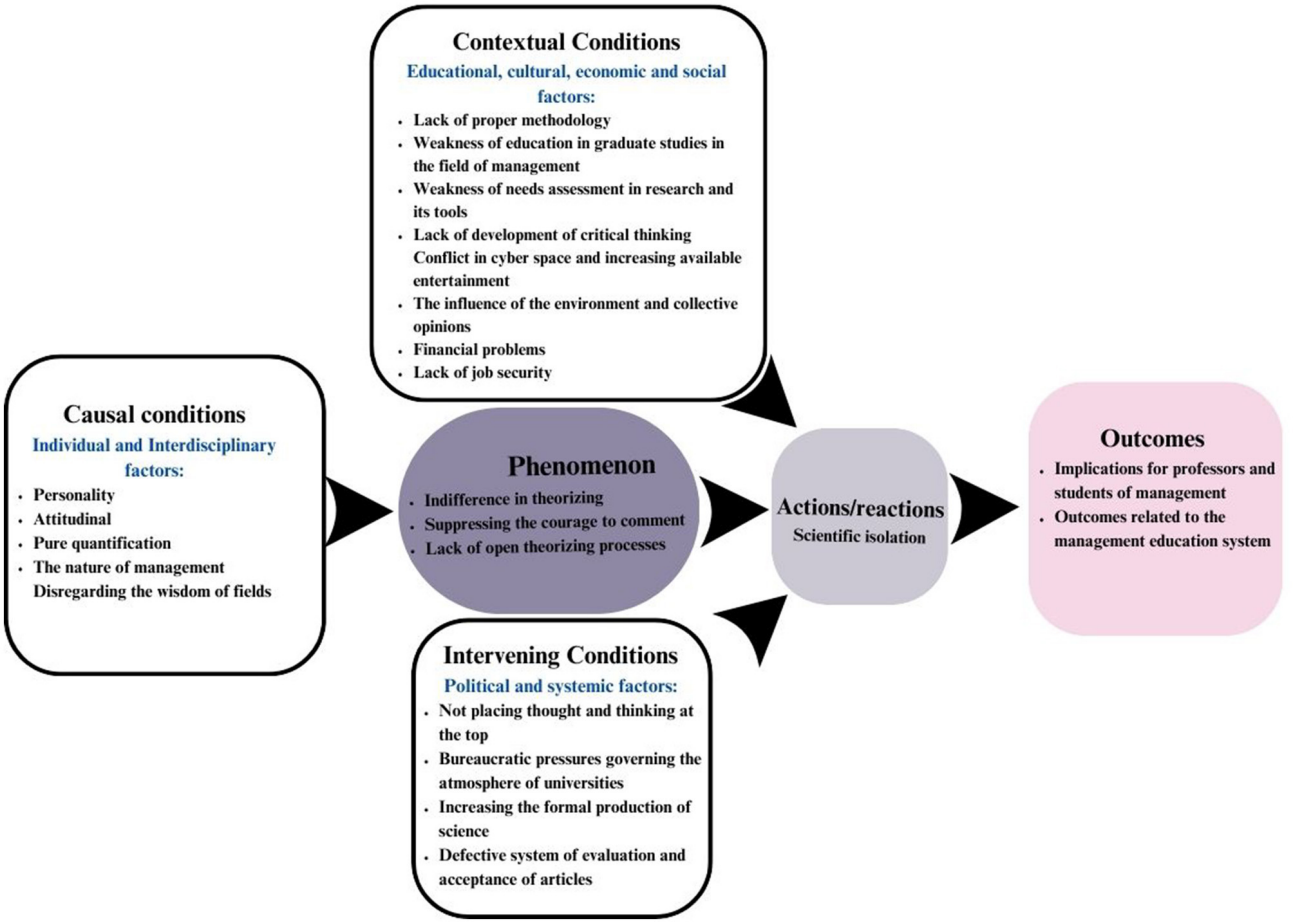
Axial coding.

Axial coding is the second stage of analysis in foundational data theorizing. The purpose of this stage is to establish a relationship between the classes produced in the open coding stage. This study was based on the paradigm model, which helped the theorist in the theorizing process. The axial coding based on the axial model is presented in Figure 1.

### 3.3 Third step: selective coding and theory creation

“Selective coding” is the process of integrating and improving categories. In this stage of coding, the database theorist writes a theory on the relationships between the categories in the axial coding model. At a basic level, this theory provides an abstract explanation of the process being studied in the research. The process of integrating and refining theory in selective coding (Straus, 1987; Lee, 2001, p. 50) involves techniques such as writing a storyline that connects categories, as well as the process of categorization through personal notes on theoretical ideas (Creswell, 2015, p. 398).

### 3.4 Narrative of research: indifference theory in theorizing in management studies

This study addresses the question of which theory explains indifference in theorizing in management research. Based on the analysis of the data collected from the interviews, a paradigm model, as shown in Figure 1, was finally developed. Indifference, a key concept in the presented model, represents a type of scientific silence, indicating the unwillingness or inability of researchers to create new and effective theories in the field of management. As shown in the figure, the causal conditions, such as individual and in-field factors, influence the core category (indifference in theorizing). Along with the contextual conditions and intervening factors, these lead to scientific isolation, which ultimately affects professors and students in the management field and is related to the educational system within this field. In the following section, the main categories and their components are explained.

The causal conditions that contribute to the lack of theorizing in the management field in Iran include two main categories of individual and intra-discipline factors. The human-centered nature of research subjects and their complexities, especially the complexity of organizations in management research, has narrowed the scope for theorizing. The field of management is an applied field, so in the production of its knowledge, there is a close link between the researcher and the subject under study. The subject studied by researchers in the field of management is the organization and its constituent element, the human factor. This cannot be easily placed on a study table, analyzed, and combined. Researchers must enter the organization and obtain permission to do so. Even filling out a questionnaire requires permission from the security organization in the public sector. Therefore, the phenomenon tested in the management field cannot be grasped and analyzed immediately. Even many Iranian organizations prevent researchers from entering the organization. Based on this, if researchers in management are to focus more on this issue, the slow pace of scientific production is inherent to the field of management (Danaeifard, 2009). Unfortunately, the fields of humanities are neglected in the country and receive little attention. While the country's officials are willing to invest in everything, they are not willing to invest in the humanities because its value is not clearly understood. This issue was also not clear in the West, but through constant self-criticism, the West came to recognize the importance of the humanities. The atmosphere in our society is not conducive to the humanities, which is why excellent students do not pursue humanities fields, and sensitive jobs are less likely to be held by humanities graduates. Another reason for the neglect of the humanities is the lack of recognition of their importance in culture-building. The problem has been compounded by officials who, upon taking office, did not have a clear understanding of the priorities of the humanities. In fact, some individuals made decisions about the humanities without truly understanding why these fields are important. The key issue is that we tend to view the sciences solely through the lens of expertise. The humanities play an important role in the development of the experimental sciences. They prepare individuals to fulfill their citizenship duties regarding social responsibilities, allow them to get acquainted with creative ideas outside their field, help them foster the skill of self-criticism, and enhance their ability to communicate and cooperate with others. In addition, with the help of the humanities, individuals can observe the impact of science and technology on society (Golshani, 2016).

In addition to the influence of individual and intra-discipline factors on theorizing, the existence of contextual conditions, such as educational, cultural, social, and economic factors, seems necessary to create a suitable platform for researchers. Without proper information and education, one cannot master the research method. Information and education are like firewood ready to ignite the process of theorizing (Bagheri, 2009). Unfortunately, by examining the educational factors, it became clear that we are facing a shortage of management knowledge and a lack of suitable educational programs within the country. The main reason for that is the dominance of traditional education methods, especially in post-secondary education. If appropriate education and training programs on the philosophy of science are prioritized in universities, the minds of management students and researchers will be better equipped to build theories. In addition, our findings indicate that an excessive focus on problem-solving can create limitations and gaps in achieving a deeper understanding of the challenges within management systems. One of the participants stated that “*we should shift our research toward problem-oriented research*.” In this regard, we point out that while problem-oriented research can help improve the current situation, it is crucial to consider the theoretical and philosophical contexts. However, it is important to focus on creating a balance between theory and practice in such a way that sufficient theoretical space is ensured for the development of new ideas and practical challenges are answered. The cultural factors that govern academic environments, including the culture of xenophobia, the sacredness of certain texts, and undue prejudice against some scientific figures, have led researchers to close their minds to knowledge and even emerging scientific voices. On the other hand, in the field of management, it is common for foreign sources to be preferred over domestic sources, and many do not believe that a person from Iran could have contributed an idea, conceptual framework, and foundational theory (Danaeifard, 2009). In this way, strong norms within universities and scientific circles can have negative consequences. These norms can stifle innovation (Sumpter et al., 2021). They can prevent members of society from staying open to new ideas and from recognizing and responding to strategic changes in the environment (Giorgi et al., 2015). In addition, the belief that theorizing is a difficult task and can only be undertaken by experienced and senior researchers is also considered to be a fallacy that keeps young researchers away from theorizing.

The social factors that govern society are very important in the construction of theory. The individual and social life of humans, along with all human works, artifacts, and social rules, exist within the context of the society and its social and cultural values. The interventions, interactions, and even conflicts within this context affect the individual and social affairs of humans. By understanding this and its effects, it becomes clear that science is a cultural phenomenon, and values (both scientific and non-scientific) shape and influence the researcher's choice of topics to study (Bagheri, 2009). Therefore, holding critique and debate sessions, Delphi sessions, and brainstorming activities in universities can lead to discussion and exchange of opinions between professors and students and give them the opportunity to think more critically. On the one hand, the introduction of new technologies and the bombardment of information through various social networks have caused confusion among researchers as identifying useful information and data from the vast amount of available content has become increasingly difficult for them. In addition, the overwhelming presence of virtual space and new entertainment, with their unique attractions and availability, has distracted students from concentrating. Another factor that influences the context is the economic factor. As mentioned in Maslow's hierarchy of needs, livelihood is one of the most basic and fundamental human needs. As long as a person has livelihood and economic concerns, he cannot focus on other aspects of his life, such as self-fulfillment. Unfortunately, in recent years, we have seen that due to sanctions and rampant inflation, the economic problems of the majority of people in society have increased. In particular, professors who are part of the middle class have been significantly affected by these issues.

Therefore, the quality of research work is affected by such problems. Graduate students have also lost their motivation to engage in scientific research due to job concerns and a lack of financial support from universities. Economic security helps individuals conduct research without having to worry about their livelihood, enabling them to make significant contributions to scientific production. Among the mentioned factors, some intervening factors such as political and systemic factors have also contributed to the decline and lack of theorizing in the field of management. Science should not be subservient to politics. Theorizing is never done with orders and directives, and it requires its own context and environment. Unfortunately, the flawed system governing the universities often leads researchers to focus on increasing the quantity of scientific productions, rather than improving their quality. In the field of management studies, the purpose of conducting research is often to increase the number of articles, rather than fostering a deeper intellectual connection with the findings (Danaeifard, 2008). In addition, the disproportion between the number of students and professors discourages people from showing enthusiasm toward conducting quality research and theorizing, which are time-consuming. Unfortunately, there is a lack of communication between management professors and students and organizations and businesses, as well as a failure to create practical spaces for applying management science. In the upstream documents outlining the transformation of the country's higher education and research system, emphasis has been placed on the effective connection between universities and research centers and industry and related sectors of society, as well as on the achievement of the required advanced technologies. The government, university, and industry communication chain is recognized as the facilitator, producer, and end-user of research and knowledge. The relationship among university institutions, industry, and the government is one of the most essential in any society as it supports the growth and prosperity of these institutions, as well as the advancement and improvement of societal conditions. The experience of different countries indicates that the creation and success of such a relationship have been important factors in their social, cultural, and economic growth and development (University of Science and Technology, 2017).

The core category (indifference in theorizing), which is a subjective form of a phenomenon that is the basis of the process (Creswell, 2015, p. 398), includes two main categories: suppression of boldness in commenting and lack of open theorizing processes. Causal, contextual, and intervening factors contribute to the fear of theorizing, the promotion of adaptationism and conservatism, an addiction to common theories, a lack of scientific self-confidence, and a failure to share concepts, frameworks, and theoretical relationships. In addition, the case examples represent collective action problems that contribute to the formation of indifference in theorizing within the field of management.

Finally, based on the causal conditions affecting the nuclear category, as well as the contextual and intervening factors mentioned above, we observed scientific isolation as an action, which actually indicates research indifference, reduced research interactions, and the separation of professors and students from the scientific community of management. Its outcomes are related to both the professors and students in the field of management, as well as to the educational system within the management field. When a person reaches intellectual stagnation, they lose their spirit and commitment to theorizing and become either bored with it or alienated within a failing system.

### 3.5. Quality and accuracy

This research was studied and reviewed by three professors. External reviewers, such as the participants in this study, who assess the data-based theory using the criteria of good science, may demonstrate that the theory includes valid and reliable data (Creswell, 2015). To ensure the reliability of the research, at each stage of the data collection and analysis, the derived categories were shown to the interviewees to confirm the accuracy of the content. After the formation of the theory, the prepared paradigm model was presented to all these individuals, allowing them to suggest changes, removals, or modifications. Ultimately, their feedback was incorporated.

### 3.6 Theoretical theorems

Theorem 1: Individual and intra-discipline factors such as the personality and attitude of people, mere quantification, the nature of the humanities, and the neglect of disciplinary wisdom contribute to indifference in theorizing.

Theorem 2: The suppression of boldness in expressing opinions and the lack of open theorizing processes lead to scientific isolation.

Theorem 3: Educational, cultural, economic, and social factors such as a lack of appropriate methodology, weakness of graduate education, inadequate research needs assessment, a lack of critical thinking development, conflicts in virtual spaces, the increase in available entertainment, and the influence of the environment and opinions, as well as economic problems and job insecurity, collectively contribute to scientific isolation.

Theorem 4: Political and systemic factors such as a failure to prioritize thought and critical thinking, bureaucratic pressures governing the atmosphere of universities, the increase in the formal production of science, and the flawed system of evaluation and acceptance of articles contribute to scientific isolation.

Theorem 5: Scientific isolation is an action and interaction that leads to outcomes that are related to professors and students and the educational system within the field of management.

Theorem 6: The outcomes related to professors and students and the outcomes related to the educational system within the field of management are the results of scientific isolation. In fact, the indifference of management students and professors toward progress, a decline in the spirit of commitment to theorizing, a weakening of the research spirit, boredom with theorizing, reduced motivation, individual alienation, intellectual stagnation, and a lack of local theories of management, along with a lack of development in management in Iran and a of scientific approach to management, contribute to the weakness of management theories in Iran.

## 4 Conclusion

Theorizing is not exclusive to elite scientists or those with management experience. Theory building is a technical skill that can be learned and applied (Shepherd and Suddaby, 2017). Therefore, the purpose of the current research was to explain the indifference model in theorizing within management research using a grounded theory approach. By identifying the challenges and obstacles faced by management researchers, it aimed to demonstrate that by removing some obstacles, it is possible to make an effective contribution to the production of management science in Iran.

Management is now one of the developing fields that relies heavily on theoretical foundations. Theory-oriented research can provide an effective foundation and support for the progress and improvement of management practice, so to progress in management practice, we must develop theory and apply it properly in the country. Some managers, driven by their mistrust and disbelief in theory, believe that facts and theories are diametrically opposed. They view facts as true and accurate, but consider theories as nothing more than unrealistic speculation. Management is both an art and a science of applying knowledge to administrative and organizational problems It aims to describe, explain, and analyze the various behaviors of people in different organizational roles and levels, offering a deeper understanding of the daily challenges faced in management and organization and providing solutions.. Theoretical solutions are required for scientific problems. As a result, by applying appropriate theories, the manager can identify the source of the problems and propose hypotheses for improving performance. Theory, as a methodical process, can provide an analysis of the successes and failures of various programs, preventing managers from using the “trial and error” method. The manager must use theory as a symbolic construction.

On the other hand, scientific silence is one of the serious challenges in management research, which can lead to theoretical isolation and an inability to produce new knowledge. Due to the lack of expression of new theories and ideas, this phenomenon reduces the diversity and richness of science and helps fuel indifference in theorizing. Researchers may give up their ideas due to the fear of rejection or non-acceptance of their ideas. This silence intensifies, especially in educational processes and interactions between professors and students, and promotes a culture in which criticism and examination of ideas are marginalized. Therefore, emphasizing the removal of existing obstacles to expressing the theories and experiences of researchers can help improve the theorizing processes and, in general, scientific development in the field of management. Given that the country is facing challenges in theorizing within the field of management, it is proposed to end the problems of collective action by reducing the bureaucratic pressures that govern the atmosphere of universities, increasing attention to critical thinking, and encouraging managers and students to share concepts, frameworks, and theoretical relationships. It is also suggested that fundamental changes be made to the country's structure and education system, particularly at the university level, as some education systems, rather than instilling in managers a spirit of questioning and criticism, train them to be one-dimensional and pragmatic. Managers who are products of such systems, in most cases, either have no theoretical support for their activities and decisions or they theorize based on their own imaginations and simply follow a pattern.

While this study was conducted in Iran, we recognize that our findings on the theorization of indifference may be applicable in various cultural and educational contexts. Thus, we propose future research to explore the potential for further expansion of this study. The researchers in the present study, focused on Iran, faced limitations such as limited access to professors, difficulty conducting interviews at different universities across the country, and the non-response and refusal of some professors to participate in the interviews. Today, management is one of the developing fields that increasingly relies on the development of theoretical foundations. Theory-oriented research can provide an effective basis and support for the progress and improvement of management practice. Therefore, to advance in management practice, it is essential to develop a theory and ensure its proper application in the country.

## Data Availability

The datasets presented in this article are not readily available because the first author should be contacted with a reasonable request for the data. Requests to access the datasets should be directed to maryam.asgharinajib@semnan.ac.ir.
